# CAN CHATGPT IDENTIFY AND MANAGE FALL RISKS IN OLDER ADULTS?

**DOI:** 10.1093/geroni/igae098.2987

**Published:** 2024-12-31

**Authors:** Huai Cheng

**Affiliations:** Minneapolis VA Hospital, Playmouth, Minnesota, United States

## Abstract

Fall is one of the most common geriatric syndromes in older adults. Identifying and managing fall risks in older adults is very important but can be time-consuming. ChatGPT can answer complex questions such as passing USMLE and clinical questions. In this study, the author prompted ChatGPT to identify and manage fall risks in one old adult via a clinical vignette from a published curriculum and test whether ChatGPT can be used as a screening and management tool in daily practice. ChatGPT was prompted to respond to this clinical vignette with three approaches. First, ChatGPT was prompted to answer one general question on fall risks in old adults; Second, ChatGPT was prompted to answer 5 pre-test questions on fall prevention in older adults; Last, ChatGPT was prompted to answer 5 questions based on this clinical vignette. The author was the only person to determine the correctness of output from ChatGPT. ChatGPT can provide a comprehensive summary of 10 common fall risks in older adults and correctly answer 5 out of 5 pre-test questions with only a few answers missing. For a clinical vignette, ChatGPT provided correct answers on all five questions with only a few answers missing. ChatGPT can be used as a screening and management tool in daily practice.

